# Endothelial progenitor cells and neural progenitor cells synergistically protect cerebral endothelial cells from Hypoxia/reoxygenation-induced injury via activating the PI3K/Akt pathway

**DOI:** 10.1186/s13041-016-0193-7

**Published:** 2016-02-03

**Authors:** Jinju Wang, Yusen Chen, Yi Yang, Xiang Xiao, Shuzhen Chen, Cheng Zhang, Bradley Jacobs, Bin Zhao, Ji Bihl, Yanfang Chen

**Affiliations:** Department of Pharmacology & Toxicology, Boonshoft School of Medicine, Wright State University, 3640 Colonel Glenn Hwy, Dayton, OH 45435 USA; Department of Neurology, Affiliated Hospital of Guangdong Medical College, Zhanjiang, 524001 Guangdong China; Wuhan Institute of Physical Education, College of Health Science, Wuhan, 430079 Hubei China; Department of Neurology, Wright State University, 3640 Colonel Glenn Hwy, Dayton, 45435 Ohio USA; Department of Internal Medicine, Wright State University, 3640 Colonel Glenn Hwy, Dayton, 45435 Ohio USA

**Keywords:** EPCs, NPCs, Cerebral ECs, Co-culture, PI3K/Akt signal pathway, Hypoxia-reoxygenation injury, VEGFR2, VEGF, BDNF

## Abstract

**Background:**

Protection of cerebral endothelial cells (ECs) from hypoxia/reoxygenation (H/R)-induced injury is an important strategy for treating ischemic stroke. In this study, we investigated whether co-culture with endothelial progenitor cells (EPCs) and neural progenitor cells (NPCs) synergistically protects cerebral ECs against H/R injury and the underlying mechanism.

**Results:**

EPCs and NPCs were respectively generated from inducible pluripotent stem cells. Human brain ECs were used to produce an in vitro H/R-injury model. Data showed: 1) Co-culture with EPCs and NPCs synergistically inhibited H/R-induced reactive oxygen species (ROS) over-production, apoptosis, and improved the angiogenic and barrier functions (tube formation and permeability) in H/R-injured ECs. 2) Co-culture with NPCs up-regulated the expression of vascular endothelial growth factor receptor 2 (VEGFR2). 3) Co-culture with EPCs and NPCs complementarily increased vascular endothelial growth factor (VEGF) and brain-derived neurotrophic factor (BDNF) levels in conditioned medium, and synergistically up-regulated the expression of p-Akt/Akt and p-Flk1/VEGFR2 in H/R-injured ECs. 4) Those effects could be decreased or abolished by inhibition of both VEGFR2 and tyrosine kinase B (TrkB) or phosphatidylinositol-3-kinase (PI3K).

**Conclusions:**

Our data demonstrate that EPCs and NPCs synergistically protect cerebral ECs from H/R-injury, via activating the PI3K/Akt pathway which mainly depends on VEGF and BDNF paracrine.

## Background

Brain endothelial cells (ECs) are critical components of the blood brain barrier (BBB). Increased BBB permeability leads to the development of tissue swelling, inflammatory cell infiltration and subsequently exaggerate injury in ischemic stroke [1]. Therefore, protection of ECs and BBB function should be an important strategy for reducing ischemic injury. On the other hand, endothelial progenitor cells (EPCs) have been suggested to participate in EC protection, repair and angiogenesis [2]. Transplantation of EPCs is a promising cell therapy for ischemic diseases such as acute myocardial infarction and stroke [3–5]. Our previous studies have shown that EPC infusion promotes angiogenesis in mouse ischemic stroke models [5, 6]. EPCs released angiogenic growth factors, such as vascular endothelial growth factor (VEGF) and insulin-like growth factor, could be responsible for the beneficial effect of EPC conditioned medium on the viability of H_2_O_2_-compromised human umbilical vein ECs [7, 8]. Currently, we do not know whether EPCs can protect cerebral ECs against hypoxia/reoxygenation (H/R)-injury.

Transplantation of neural progenitor cells (NPCs) has also been shown to be effective for treating ischemic stroke in animal models [9, 10]. In addition to generating neurons, grafted NPCs could promote angiogenesis in a rodent stroke model [11]. A recent report suggests that co-culture with NPCs decreases the passive permeability of brain ECs [12]. Collectively, these studies indicate a crosstalk between NPCs and ECs. However, it is unclear whether NPCs and EPCs have synergistic effects on EC protection.

The PI3K/Akt signal pathway participates in various cellular processes such as cell survival and proliferation [13]. Previous studies have shown that activation of the PI3K/Akt signal pathway promotes neuron survival [14, 15], cardiac microvascular EC migration [16], and axonal outgrowth compromised by oxygen-glucose deprivation [17, 18]. It is unknown whether this pathway is involved in the mechanism of the benefits of NPCs and EPCs.

The aims of this study were to elucidate whether EPCs and NPCs synergistically protect brain ECs from H/R-induced injury and to explore whether the effects are mediated by the PI3K/Akt signal pathway.

## Results

### NPCs and EPCs were successfully generated from human inducible pluripotent stem cells

**Fig. 1:**
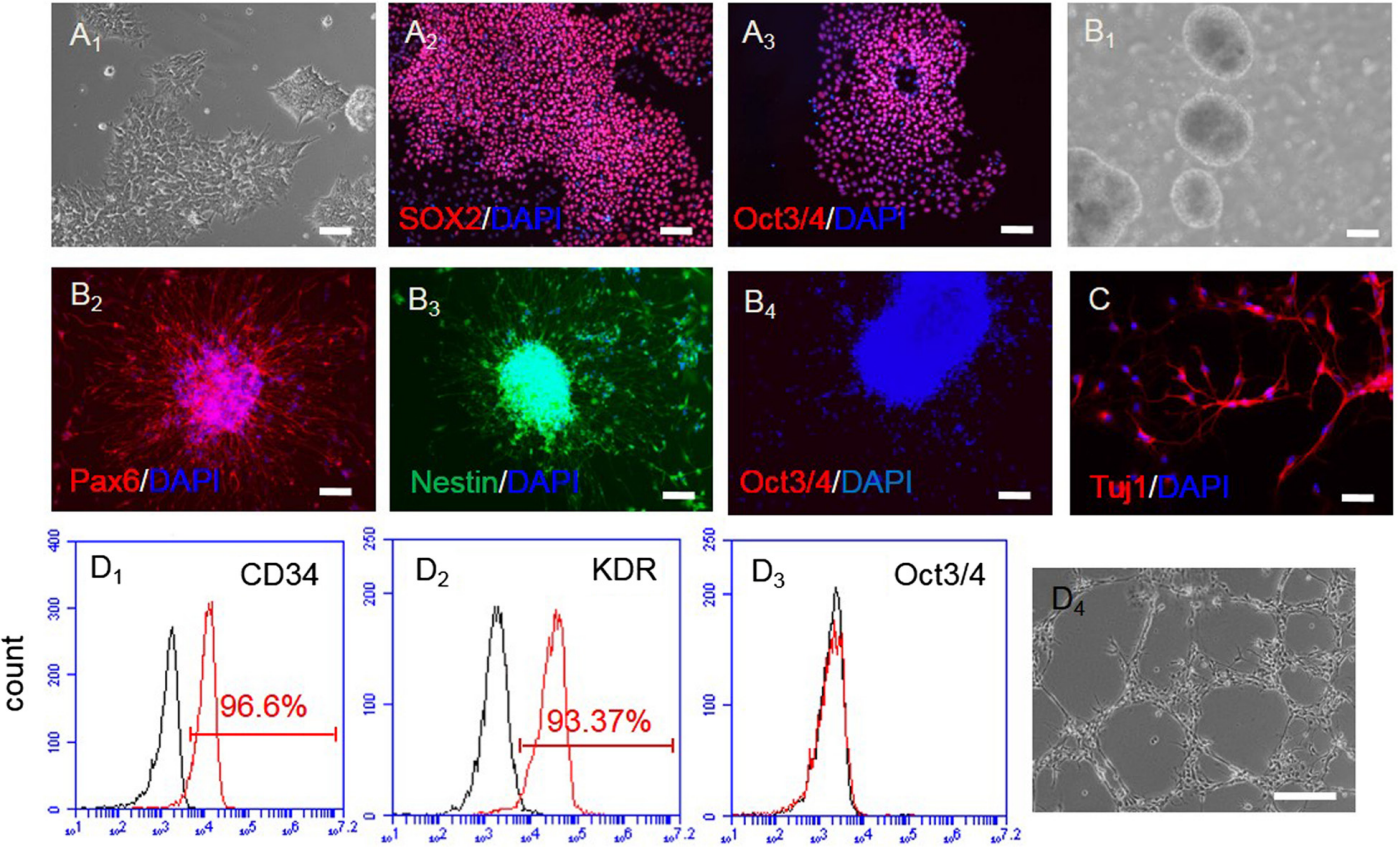
Characterization of EPCs and NPCs from human iPSCs. A1, iPSCs grew on feeder-free matrigel. Scale bar: 2000 μm. A2-A3, iPSCs expressed Sox2 and Oct3/4. B1, NPCs grew as neurospheres after 7 days neural differentiation. B2-B4, generated NPCs positively expressed neural progenitor markers nestin and Pax6, and were negative for pluripotent stem cell marker Oct3/4. C, generated neurons positively stained neuron specific marker Tuj1. D1-D3, purified EPCs positively expressed endothelial progenitor markers CD34 and KDR, but were negative for Oct3/4. Black curves: isotype control; red curves: antibodies. D4, tube formation of EPCs. DAPI counterstained cell nucleus. Scale bar: 200 μm from A2-D4

As shown in Fig. 1, the human inducible pluripotent stem cells (iPSCs) grew as colonies staining positively for pluripotent markers, Sox2 and Oct3/4. The generated NPCs grew as neurospheres after 7-day neural induction, and expressed neural progenitor markers pax6 (98 ± 1 %) and nestin (96 ± 1.5 %), but not expressed Oct3/4, indicating a high differentiation efficacy. The generated NPCs had ability of differentiating into neurons, which was evidenced by expressing neuron specific marker Tuj1.

After 7-day EPC induction, approximately 48 ± 2.1 % of cells positively expressed endothelial progenitor marker CD34. In order to get a pure population of EPCs, we used CD34-conjugated microbeads to enrich the generated EPCs. The CD34-conjugated microbeads purified cells positively expressed CD34 (96 ± 2.1 %) and KDR (95 ± 1.8 %). As expected, the purified EPCs did not express Oct3/4. In addition, the generated EPCs had tube formation ability as revealed by matrigel assay.

### Co-culture with EPCs and NPCs synergistically protected ECs from H/R-induced apoptosis and compromised viability via activating the PI3K pathway

**Fig. 2:**
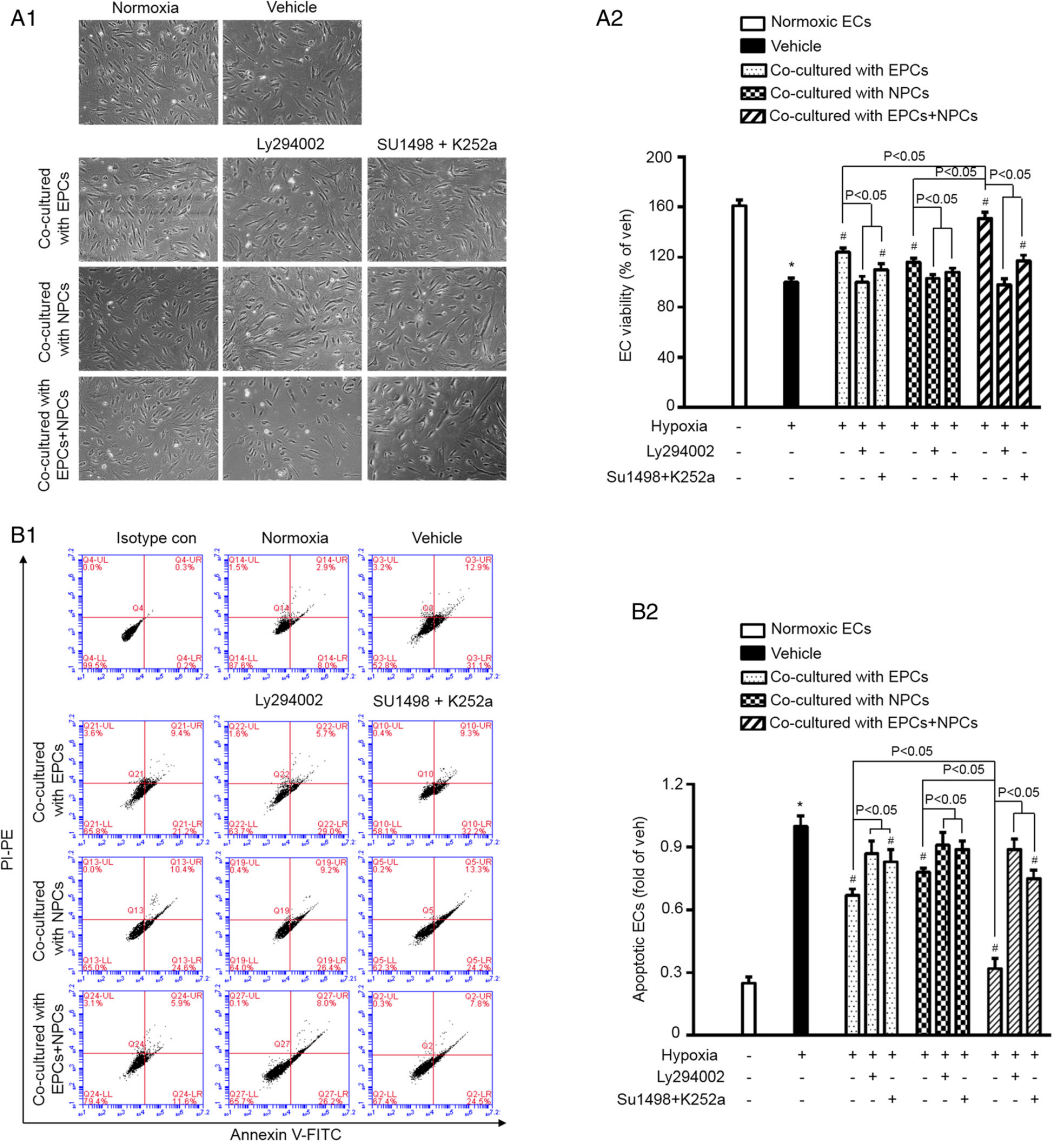
EPCs and NPCs promoted the survival of H/R-injured ECs via activating the PI3K pathway. MTT assay and PI/FITC-Annexin V apoptosis assay were conducted on H/R-injured ECs co-cultured with EPCs and/or NPCs for 24 h as described in Material and Methods. A1, representative morphology images showing the viability of ECs. A2, summarized data showing EC viability which is synergistically increased when co-cultured with the combination of EPCs and NPCs than that co-cultured with EPCs or NPCs alone. B1, representative flow plots of EC apoptotic rate. B2, summarized data of the apoptotic rate of ECs, showing that the combination of EPCs and NPCs offers better anti-apoptotic effect than EPCs or NPCs alone. Block the PI3K pathway could diminish the beneficial effects of EPCs and/or NPCs. And the PI3K pathway upstream blockers, SU1498 and K252a, reduced these effects of EPCs and NPCs. *p < 0.05, vs. Normoxia; #*p* < 0.05, vs. vehicle. Data are expressed as mean ± SEM, *n* = 6/group/measurement. LY294002: PI3K inhibitor; SU1498: VEGFR2 inhibitor; K252a: TrkB inhibitor

After exposed to the hypoxic condition for 6 h, ECs were co-cultured with EPCs and/or NPCs for 24 h, followed with apoptotic assay or MTT assay. Results (Fig. 2) showed that co-culture with EPCs and NPCs exerted a greater effect on decreasing H/R-injured EC apoptosis than that co-culture with EPCs or NPCs separately did (vehicle vs. EPCs or NPCs, *p* < 0.05; EPCs and NPCs vs. EPCs or NPCs, *p* < 0.05). Similarly, the EC viability was also synergistically increased by co-culture with EPCs and NPCs (vehicle vs. EPCs or NPCs, *p* < 0.05; EPCs and NPCs vs. EPCs or NPCs, *p* < 0.05). The synergistic effects on reducing EC apoptosis and improving EC viability were achieved by an increase of approximately 24 and 28 %, respectively.

Moreover, our data showed that the PI3K inhibitor (LY294002) pre-treatment could completely abolish the abovementioned effects of EPCs and/or NPCs, suggesting that the beneficial effects of EPCs and NPCs are mediated by the PI3K pathway. To define the contribution of VEGFR2 and TrkB (PI3K upstream molecules) to these effects, the respective inhibitors SU1498 and K252a were pre-added in the co-culture system. Our results revealed that blockade of the VEGF/VEGFR2 and BDNF/TrkB signals reduced the effects of EPCs and NPCs.

### Co-culture with EPCs and NPCs synergistically decreased the oxidative stress of H/R-injured ECs via activating the PI3K pathway

**Fig. 3:**
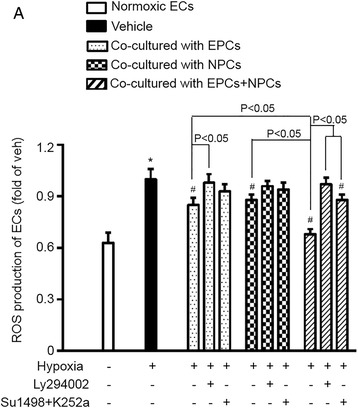
EPCs and NPCs decreased ROS production via activating the PI3K pathway. A, ROS production, showing that ROS over-production was much decreased in H/R-injured ECs co-cultured with EPCs and NPCs than that co-cultured with EPCs or NPCs alone. LY294002 could diminish the anti-oxidative effect of EPCs and/or NPCs, and the combination of SU1498 and K252a reduce such effect. *p < 0.05, vs. Normoxia; #*p* < 0.05, vs. Vehicle. Data are expressed as mean ± SEM, *n* = 6/group/measurement. LY294002: PI3K inhibitor; SU1498: VEGFR2 inhibitor; K252a: TrkB inhibitor

As shown in Fig. 3, ROS production was decreased in H/R-injured ECs co-cultured with EPCs or NPCs (vehicle vs. EPCs or NPCs, *p* < 0.05). Moreover, co-culture with EPCs and NPCs decreased ROS production to a larger extent than that with EPCs or NPCs alone did (EPCs and NPCs vs. EPCs or NPCs, *p* < 0.05). The synergistic effect on decreasing ROS production was obtained by about 18 % increase.

As expected, pre-treatment with PI3K inhibitor, LY294002, abolished the anti-oxidative effect of EPCs and/or NPCs on H/R-injured ECs. Pre-treatment with a combination of the PI3K upstream blockers SU1498 and K252a reduced the most anti-oxidative effects of EPCs and NPCs on H/R-injured ECs. All of these data indicate that the anti-oxidative effect of EPCs and NPCs is mediated by the PI3K signal pathway.

### H/R-compromised tube formation ability of ECs was synergistically improved by co-culturing with EPCs and NPCs via activating the PI3K signal pathway

**Fig. 4:**
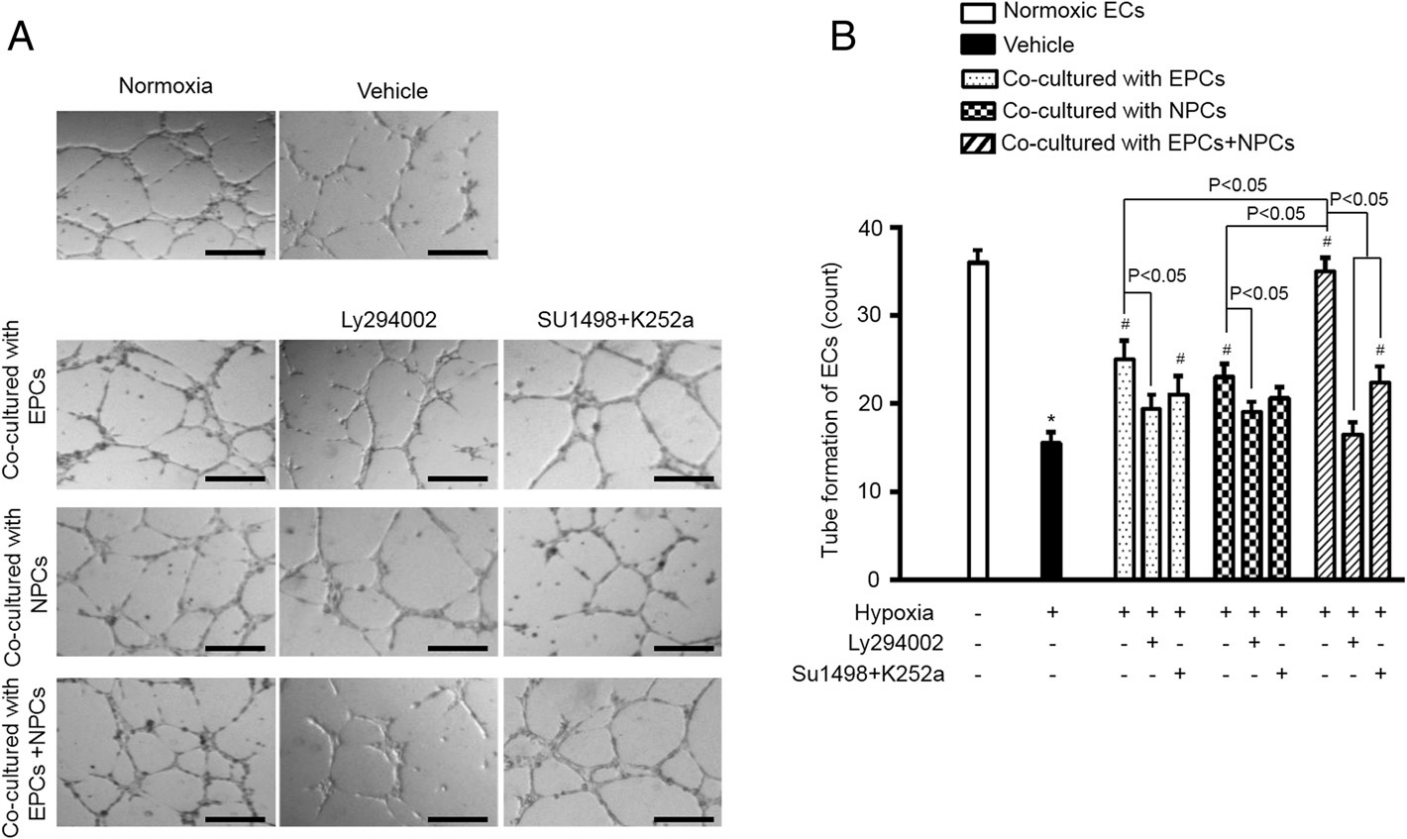
EPCs and NPCs improved the angiogenic function of H/R-injured ECs via activating the PI3K pathway. **a**, representative plots of tube formation. Scale bar: 200 μm. **b**, summarized data of EC tube formation, showing that EPC and NPC co-culture offers synergistically effects on improving EC function compared to EPCs or NPCs alone. And such synergistic effect could be blocked by LY294002, or partially abolished by the combination of SU1498 and K252a. *p < 0.05, vs. Normoxia; #*p* < 0.05, vs. Vehicle. Data are expressed as mean ± SEM, *n* = 6/group/measurement. LY294002: PI3K inhibitor; SU1498: VEGFR2 inhibitor; K252a: TrkB inhibitor

We further assessed whether co-culture with EPCs and/or NPCs altered the tube formation function of ECs exposed to H/R. The results (Fig. 4) showed that EPCs or NPCs alone increased the tube formation ability of H/R-injured ECs (vehicle vs. EPCs or NPCs, *p* < 0.05). Moreover, co-culture with EPCs and NPCs exhibited a synergistic effect on improving the tube formation ability of ECs compromised by H/R (EPCs and NPCs vs. EPCs or NPCs, *p* < 0.05). The synergistic effect of EPC plus NPC co-culture on improving the tube formation ability was increased by approximately 19 %.

In order to elucidate the possible role of the PI3K pathway in the effect of EPCs and/or NPCs on EC tube formation, the pathway specific inhibitor LY294002 was used in the co-culture study. Our results showed that PI3K inhibition entirely abolished this effect of EPCs and NPCs. Similarly, to further explore whether VEGF/VEGFR2 and BDNF/TrkB signals could be responsible to trigger the activation of the PI3K pathway, we pre-added their respective inhibitors SU1498 and K252a into the co-culture system. As we expected from the data of apoptotic and MTT assays, blockade of the VEGF/VEGFR2 and BDNF/TrkB signals reduced the effect on tube formation.

### The endothelial permeability was improved by co-culturing with EPCs and NPCs

**Fig. 5:**
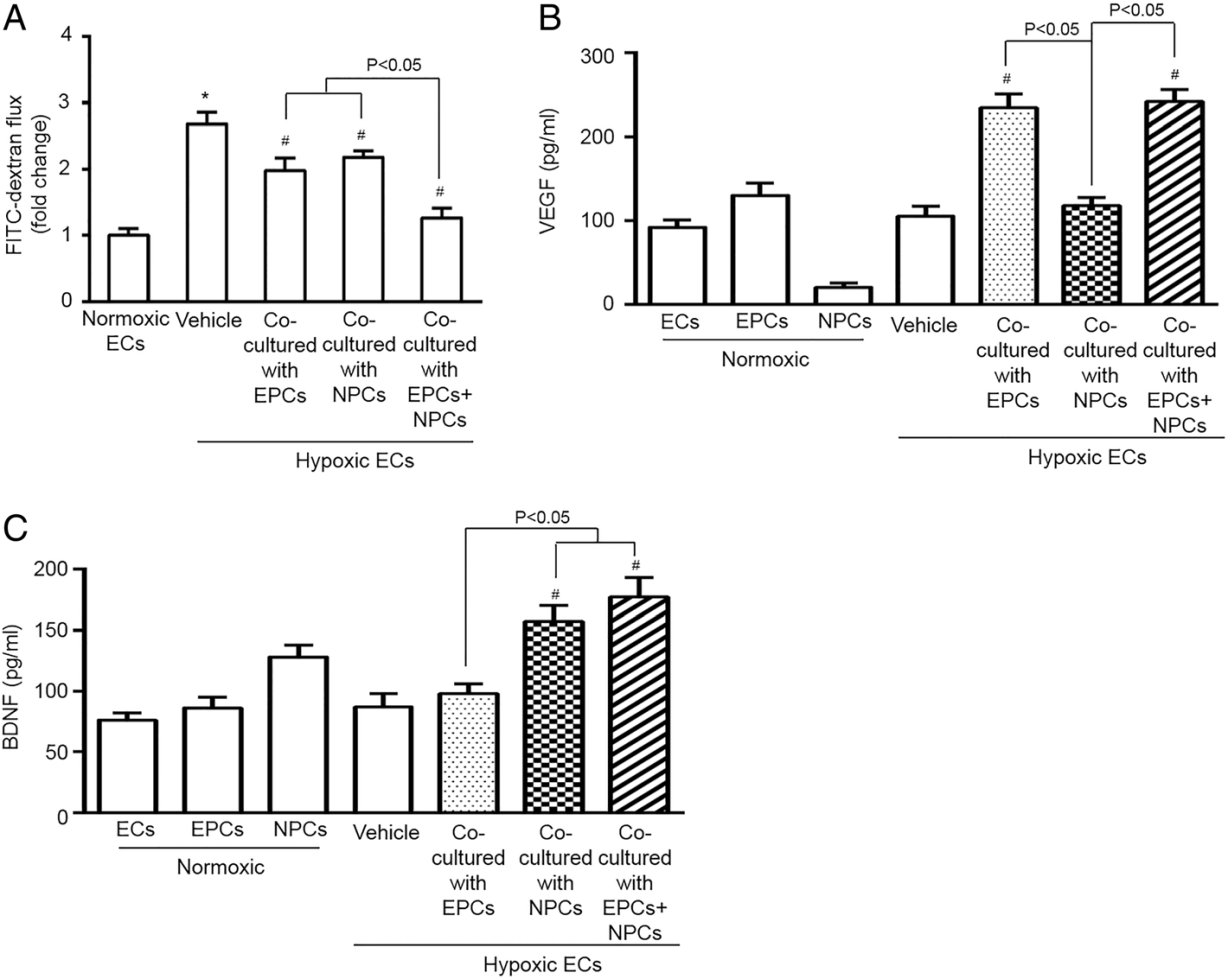
EPCs and NPCs modulated the permeability and VEGF and BDNF secretion of H/R-injured ECs. **a** fold change of FITC-dextran flux, showing that the combination of EPCs and NPCs has better effects than EPCs or NPCs alone on improving the endothelial barrier function of H/R-injured ECs. **b**, **c** the levels of VEGF and BDNF in culture medium of normoxic cultured ECs, EPCs and NPCs, as well as hypoxic ECs co-cultured with EPCs, or NPCs, or both EPCs and NPCs. The summarized data showing that the levels of VEGF and BDNF were much increased in H/R-injured ECs co-cultured with EPCs and NPCs than that co-cultured with NPCs or EPCs alone. *p < 0.05, vs. Normoxia, #*p* < 0.05, vs. vehicle. Data are expressed as mean ± SEM, *n* = 6/group/measurement

Under physiological conditions, the endothelial membrane is impermeable to macromolecules (mass weight around 70 k Dalton) [19]. We performed permeability assay to evaluate whether co-culture of EPCs and/or NPCs could improve the barrier function of ECs compromised by H/R. As expected, H/R injury increased trans-endothelial permeability to FITC-conjugated dextran (mass weight around 10 k Dalton). Co-culture of EPCs or NPCs decreased the flux of FITC-dextran, and EPCs combined with NPCs was more effective in decreasing the FITC-dextran flux through the EC monolayer (Fig. 5a).

### Co-culture with EPCs and NPCs complementarily elevated the levels of VEGF and BDNF in the conditioned medium of ECs exposed to H/R

In order to explore the mechanisms underlying the protective benefits of EPCs and NPCs, we performed ELISA assay to determine the levels of VEGF and BDNF in the culture medium. As shown in Fig. 5b, c, we found that co-cultured with EPCs alone increased the VEGF level, but not the BDNF level in the EC culture medium, whereas, co-cultured with NPCs alone raised the BDNF level, not the VEGF level. Moreover, co-culture with EPCs and NPCs increased the levels of both VEGF and BDNF in the EC medium, suggesting a complementary effect.

### The expression of VEGFR2 was upregulated and ratios of p-Flk1/VEGFR2 and p-Akt/Akt were increased in H/R-injured ECs co-cultured with EPCs and NPCs

**Fig. 6:**
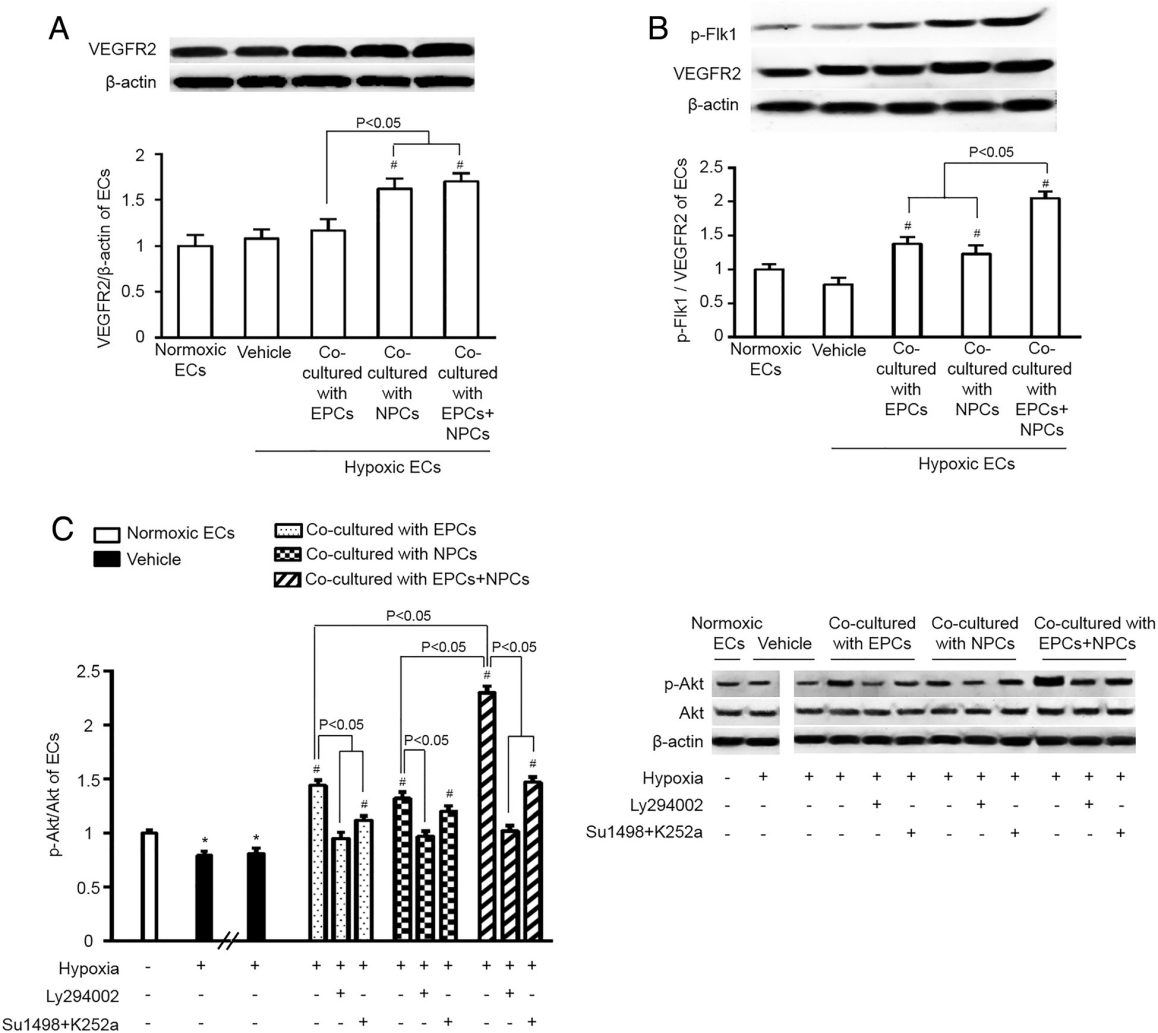
Co-culture with EPCs and NPCs activated the PI3K/Akt signal pathway on H/R-injured ECs. **a**, VEGFR2 expression was significantly upregulated in H/R-injured ECs co-cultured with NPCs or the combination of EPCs and NPCs. **b** the protein expression ratio of p-Flk1/VEGFR2 was significantly increased in H/R-injured ECs co-cultured with EPCs or NPCs, with a higher ratio in ECs co-cultured with the combination of EPCs and NPCs. **c** the protein expression ratio of p-Akt/Akt was increased in H/R-injured ECs co-cultured with EPCs and NPCs, and this effect was blocked or reduced when ECs were pre-treated with PI3K inhibitor LY294002 or VEGFR2 inhibitor SU1498 or TrkB inhibitor K252a. *p < 0.05, vs. Normoxia; #*p* < 0.05, vs. vehicle. Data are expressed as mean ± SEM, *n* = 6/group/measurement

As shown in Fig. 6a, co-culture with NPCs alone or with EPCs and NPCs similarly increased the expression level of VEGFR2 in H/R-injured ECs, whereas, co-cultured with EPCs alone did not significantly change the expression of VEGFR2 in ECs, indicating that interaction of NPCs with ECs.

Western blot results demonstrated that the expression ratios of p-Flk1/VEGFR2 and p-Akt/Akt in H/R-injured ECs were increased by co-culture with EPCs or NPCs alone, with a greater increase when co-cultured with both EPCs and NPCs (Fig. 6b, c). Data showed that the net increase of the synergistic effect on up-regulating the expression ratio of p-Akt/Akt was approximately 30 %. As expected, the PI3K inhibitor LY294002 abolished the phosphorylation of Akt, suggesting that the PI3K/Akt signal pathway is activated in ECs co-cultured with EPCs and NPCs. A combination of SU1498 and K252a decreased the phosphorylation of Akt, reflecting that it at least partially depends on the upstream molecules VEGFR2 and TrkB (Fig. 6b).

## Discussion

**Fig. 7:**
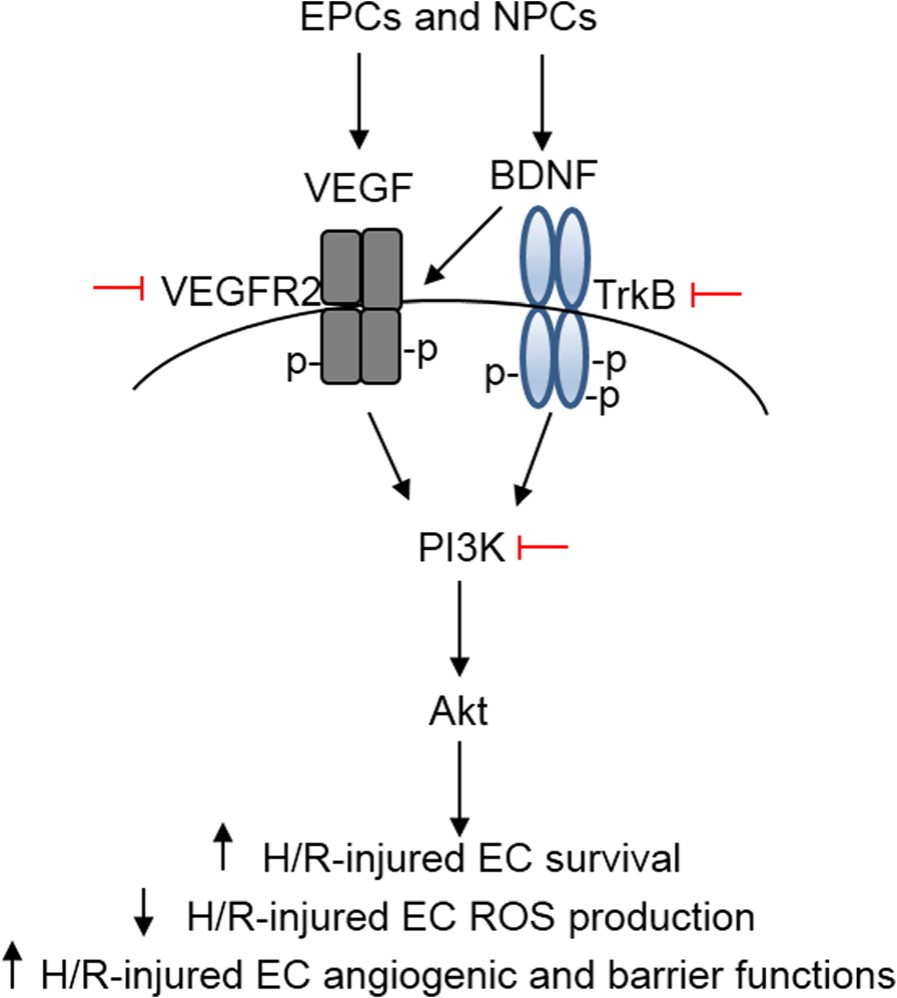
Proposed molecular mechanism for the protective effect of EPCs and NPCs on H/R-injured brain ECs. Co-culture with EPCs and NPCs synergistically increased the survival ability, decreased the oxidative stress and improved the angiogenic and barrier functions of H/R-injured EC, via activating the PI3K/Akt signal pathway that mainly depended on the progenitor paracrine (VEGF and BDNF) mediated signals. EPCs: endothelial progenitor cells; NPCs: endothelial progenitor cells; VEGF: vascular endothelial growth factor; BDNF: brain derived neurotrophic factor; VEGFR2: vascular endothelial growth factor receptor 2; TrkB: tyrosin kinase B; PI3K: phosphatidylinositol-3-kinase; H/R: hypoxia/reoxygenation; EC: endothelial cells; ROS: reactive oxygen species

In the present study, we showed that EPCs and NPCs produced from human iPSCs had synergistic beneficial effects on H/R-injured brain ECs. The major findings include: i) Co-culture with EPCs and NPCs synergistically protected ECs from H/R-induced apoptosis and dysfunction; ii) The levels of VEGF and BDNF in the medium of ECs co-cultured with EPCs and NPCs were increased; iii) Co-culture with NPCs up-regulated VEGFR2 expression and its phosphorylation on ECs; iv) Blockade of the VEGFR2 and Trkb or PI3K/Akt pathway inhibited or abolished the protective effects of EPCs and NPCs (Fig. 7).

ECs are unique and critical in maintaining normal BBB function [20]. Impairment of BBB occurs in the early stage of ischemic brain injury, leading to subsequent brain swelling and inflammatory responses [21]. Thus, protecting brain ECs from H/R-induced injury will theoretically alleviate brain tissue damage in ischemic stroke. Nevertheless, there is no clinically effective strategy to protect ECs against H/R-induced injury in acute ischemic stroke. Transplantation of stem cells has been shown to accelerate the functional recovery of ischemic stroke by promoting angiogenesis and neurogenesis [22]. Indeed, others and our studies have demonstrated that engrafted EPCs or NPCs can alleviate acute ischemic injury and promote angiogenesis and neurogenesis in an ischemic stroke mouse model [5, 6, 9, 10]. However, it is unknown whether there are synergistic effects if EPCs and NPCs are combined to treat ischemic-reperfusion stroke.

In the present study, we examined the effects of EPCs and NPCs on H/R-injured brain ECs in vitro. It is well known that iPSCs have unlimited self-renewal ability and are able to differentiate to various types of cells with less ethical issues for clinical applications [23, 24]. We successfully differentiated human iPSCs into EPCs and NPCs. To mimic the status of ECs in acute ischemic stroke, we produced an in vitro model of brain EC H/R injury, characterized with decreased viability, increased apoptosis and cellular permeability, increased ROS production, as well as compromised tube formation ability [25]. By using this model, we found that co-culture with EPCs or NPCs alone had beneficial effects on protecting ECs from H/R-induced injury, including increase in apoptosis, ROS production and intercellular permeability, and decrease in viability and capillary formation. Moreover, co-culture with both EPCs and NPCs achieved synergistic effects on those measurements by 18–28 % increase.

Numerous studies have shown that VEGF is released from EPCs and ECs, and that BDNF is released from NPCs, which are respectively responsible for the beneficial effects of EPCs and NPCs [26, 27]. In order to determine whether EPC-derived VEGF and NPC-derived BDNF are the major factors involved in the observed effects of EPCs and NPCs in this study, we have analyzed the levels of VEGF and BDNF in the culture medium of ECs. Our results showed that EPC co-culture increased VEGF, but not BDNF level in the EC medium, whereas, NPC co-culture increased BDNF, but not VEGF level in the EC medium. More importantly, the data revealed that EPCs and NPCs complementarily increased the VEGF and BDNF levels in the co-culture medium of ECs. In the present study, we did not study the dose-dependent effects of VEGF and BDNF on ECs and not compare if co-application of VEGF and BDNF is more significant than simply increasing the dose of VEGF or BDNF alone. However, our results revealed that the combination of EPCs and NPCs have synergistic effects on ECs. For exploration of the underlying mechanism, we analyzed the expression of VEGFR2 and its phosphorylation. The results showed that co-culture with both EPCs and NPCs synergistically increased the expression of p-Flk1/VEGFR2 in ECs. Of note, we found that co-culture with NPCs, but not EPCs, up-regulated the expression of VEGFR2 on ECs. This is supported by a previous report showing that BDNF increases the on a rat brain EC line [28], and suggests that NPCs can mediate the synergistic effects by its secreted BDNF and provides the basis of the synergistic effects observed in the co-culture group combining EPCs and NPCs. Furthermore, we examined the role of VEGF/VEGFR2 and BDNF/TrkB signal pathways in the beneficial effects of EPC/NPC co-culture. Our data showed that blockade of both signals largely decreased the abovementioned effects of EPCs and NPCs, suggesting that the beneficial effects of EPCs and NPCs are mainly dependent on the VEGF/VEGFR2 and BDNF/TrkB signals. These data are in consistent with the notion that VEGFR2 and TrkB are the major modulators of endothelial survival [28]. Collectively, VEGF and BDNF are the major factors for responsible of the synergistic effects of EPCs and NPCs in the co-cultures, although there are unidentified factors contributing a minor part.

The PI3K is the downstream pathway molecule of VEGFR2 and TrkB, which mediates various cell activities includes cell survival, cell proliferation [29-32]. Therefore, we conducted experiments for further pathway analysis. We found that both EPCs and NPCs increased the level of p-Akt/Akt in ECs. And there was a synergistic effect on level of p-Akt/Akt when EPCs and NPCs were simultaneously applied. The synergistic effect of EPC plus NPC co-culture was 30 % on up-regulating the protein expression ratio of p-Akt/Akt. Moreover, the protective effects elicited by EPCs and/or NPCs were abolished by blockade of PI3K with LY294002. Taken together, our data indicate that the PI3K pathway, downstream of VEGFR2 and TrkB, is responsible for the beneficial effects of EPCs and NPCs.

## Conclusion

In conclusion, our data demonstrate that EPCs and NPCs can offer synergistic benefits in protecting brain ECs against H/R injury by VEGF and BDNF paracrine-mediated activation of the PI3K/Akt signal pathway. These findings will help us to develop cell-based therapy for ischemic stroke.

## Methods

### Human iPSCs culture

The vector-free viral-free human iPSCs cell line (iPS-DF-19-9/7 T) was purchased from Wicell Research Institute (USA). The iPSCs were cultured with mTeSR1 medium (Stem cell technology) on matrigel-coated plates (BD Bioscience), and expanded every 4–5 days according to the manufacturer’s protocol. Passage of 28–44 of human iPSCs were used for NPC or EPC induction. All experimental procedures were approved by Wright State University Institutional Biosafety Committee and were in accordance with the approved guidelines.

### Generation of NPCs and EPCs from human iPSCs

EPCs and NPCs were produced from human iPSCs according to previous reports with slight modifications [14, 15]. In brief, iPSC colonies were detached with dispase (2 mg/ml in DMEM/F12; Stem cells), pooled together and cultured to form embryoid bodies (EBs) in EB medium (DMEM/F12+ 20 % knock out serum + 1 % nonessential amino acid + 0.1 mM 2-mercapethonal + 1 % penicillin-streptomycin solution) in low-attachment dishes (Corning) for 5–7 days. The DMEM medium and all supplement reagents were purchased from Gibco. The EBs were used for NPC and EPC generation.

For NPC generation, EBs were cultured in neural medium (NM: Neurobasal medium A + B27 minus Vitamin A (1x) + N2 (1x) + FGF (20 ng/ml) + EGF (20 ng/ml) + 1 % penicillin-streptomycin solution) in low-attachment dishes for 7–9 days. The neurobasal mediumA and all supplement reagents were purchased from Gibco. The generated NPCs were propagated in free-floating aggregates (neurospheres), and used for an in vitro differentiation assay to investigate neural differentiation ability [33]. The generated NPCs and neurons were confirmed by immunofluorescence analysis [34].

For EPC generation, EBs were cultured on gelatin (0.1 %) coated plates with EPC medium (EBM-2 medium + growth factor mixture + 5 % FBS + VEGFA (50 ng/ml) + FGF (25 ng/ml) for 7–9 days [14]. The EBM-2 medium and all supplement reagents were purchased from Gibco. The generated EPCs were purified by magnetic activated cell sorting (MACS) with CD34-microbeads according to the manufacture’s protocol (Miltenyi Biotec), and assessed by flow cytometry and matrigel assay.

### Characterization of generated NPCs

For immunofluorescence analysis, generated NPCs were fixed, permeabilized and blocked with blocking buffer (PBS with 1 % BSA and 1 % Triton-100), then incubated with neural progenitor specific markers nestin (1:100; Pierce), pax6 (1:50; Pierce) and pluripotent specific marker Oct3/4 (1:200; Abcam). Then the cells were incubated with Cy-3 or Alexa fluo 488-conjugated secondary antibodies (1:150; Jackson ImmunoResearch) for 2 h at room temperature. DAPI was used to counterstain nuclei.

In order to determine the neuron differentiation capability of the produced NPCs, the generated NPCs were cultured in neuron differentiation medium (NPBM medium + BDNF (20 ng/ml) + FGF (20 ng/ml) + EGF (20 ng/ml) + 1 % penicillin-streptomycin solution) for 3 weeks. The NPBM medium and all supplement reagents were purchased from Gibco. The differentiated cells were permeabilized, incubated with neuron specific marker β-tubulin (1:100; Pierce) and followed by incubation with Cy3-conjugated secondary antibody (1:150; Jackson ImmunoResearch). DAPI were used to counterstain nuclei. All images were taken under an inverted fluorescence microscope (EVOS, Life Technologies).

### Purification and characterization of generated EPCs

In order to exclude the contamination of human iPSCs, the generated EPCs were purified by using MACS according to the manufacture’s instruction. In brief, the differentiated cells (10^7^ cells) were incubated with 10 μl CD34-conjugated microbeads (Miltenyi Biotec Inc) in the refrigerator for 20 min, followed by wash in PBS for twice. The beads-binding cells were separated using an autoMACS separator (Miltenyi Biotec Inc). All CD34^+^ cells were resuspended with EPC culture medium and cultured in a regular humidified incubator.

For flow cytometry analysis, the purified EPCs were incubated with FITC-conjugated CD34, FITC-conjugated KDR, FITC-conjugated Oct3/4 or FITC-conjugated IgG for 30 min (5 μl, eBioscience) in the dark. All antibodies were purchased from eBioscience. After incubation, all samples were analyzed under flow cytometry (Accuri C6 flow cytometer). 10,000 events were collected for data analysis.

### H/R-injury model of brain ECs

Cerebral EC cell line was purchased from Cell Systems (Kirkland, WA) and cultured according to the manufacturer’s protocol. Passages 4–13 of ECs were used for experiments in this study. The H/R-injury model of ECs was produced as previously described [25]. Briefly, ECs were changed with fresh culture medium and cultured for 6 h in a hypoxic chamber (1 % O_2_, 5 % CO_2_, and 94 % N_2_; Biospherixhypoxia chamber), then reoxygenated by incubation in a standard 5 % CO_2_ incubator for 24 h. During the reoxygenation period, ECs were co-cultured with EPCs and/or NPCs as described below.

### Co-culture brain ECs with EPCs and/or NPCs

The co-culture system was set up according to a previous report with minor modifications [35]. In brief, the day before co-culture, NPCs (4 × 10^5^), or EPCs (4 × 10^5^), or NPCs (2 × 10^5^) and EPCs (2 × 10^5^) were plated into transwell membrane inserts (pore size, 0.4 μm; polycarbonate membrane, Greiner Bio-One, Germany) in NPC and/or EPC culture medium for overnight. [36] During the reoxygenation period, brain ECs (4 × 10^5^) subjected to hypoxic (1 % O_2_) were co-cultured with EPCs and/or NPCs. For signal pathway study, LY294002 (PI3K inhibitor; 20 μM, Cayman Chemical), SU1498 (VEGFR inhibitor; 5 μM, BioVision), or k252a (TrkB inhibitor; 10 μg/ml, BioVision) was added to EC culture medium 2 h prior to co-culture experiments [25, 36–38] and presented in the EC culture during the co-culture period. All inhibitors were dissolved with DMSO (Sigma) and diluted with culture medium to yield desired concentrations. ECs cultured in normoxia (5 % CO_2_, 37 °C) were used as a control. ECs in the vehicle group were cultured with EC culture medium only.

### Cell viability, apoptosis and ROS production analyses of ECs

The viability of H/R-injured ECs was measured by using a methyl thiazolyl tetrazolium (MTT) kit (Invitrogen) as we previously described with slight modification [25]. Briefly, after 24 h co-culture with EPCs and/or NPCs, the ECs culture medium was replaced with 1 ml of fresh culture medium with 100 μl of 12 mM MTT solution and incubated at 37 °C for 2 h. Then removed 850 μl of medium from the wells and added 500 μl of DMSO to each well, mixed thoroughly with the pipette and incubated at 37 °C for 10 min. Finally, transferred 100 μl of mixed solution from each well to a 96-well plate. The plate was read by a reader (Bio-teck) at 570 nm. The percent of cell viability was defined as the relative absorbance of cells in co-culture groups versus control cells. At least six wells per experiment were used in each group.

The apoptosis assay of ECs was conducted using FITC-Annexin V apoptosis detection kit (BD Bioscience) as we previously described [25]. In brief, after 24 h co-culture with EPCs and/or NPCs, the ECs culture medium was removed and rinsed twice with PBS, then de-attached with 0.25 % trypsin/EDTA, centrifuged, resuspended with 100 μl 1x Annexin V-binding buffer and incubated with 5 μl FITC-conjugated Annexin V and 5 μl propidium iodide (PI) in the dark for 15 min at RT. The stained ECs were analyzed by flow cytometry (Accuri C6 flow cytometer). The apoptotic cells were defined as Annexin V+/PI− cells. The experiment was repeated six times.

The intracellular ROS production in ECs was determined by dihydroethidium (DHE, Sigma) [25]. Briefly, after EPC and/or NPC co-culture, the EC culture medium was replaced with fresh cultured medium containing the DHE working solution (2 μM) at 37 °C for 2 h. Then the cells were detached with trypsin and were analyzed by flow cytometer (Accuri C6). The experiment was repeated six times.

ECs cultured in normoxia served as a control. ECs in the vehicle group were cultured with EC culture medium only. The synergistic effect of EPC plus NPC co-culture (Es) on ECs was calculated by using the formula: Es = (E_EPC + NPC_ − E_EPC_ − E_NPC_) / (E_EPC +_E_NPC_) x 100 %. E_EPC_ represents the effect elicited by EPC co-culture. E_NPC_ represents the effect elicited by NPC co-culture. E_EPC+NPC_ represents the effect elicited by EPC and NPC co-culture.

### Tube Formation and endothelial permeability assays of ECs

The tube formation ability of ECs was evaluated by using a tube formation assay kit (Chemicon) with slight modification [25]. After co-cultured with EPCs and/or NPCs, the ECs were trypsinized and reseed at a density of 5 × 10^3^–1 × 10^4^ onto the surface of the polymerized ECMatrix™, and incubated at 37 °C in a CO_2_ incubator for 12–16 h. The tube formation was inspected under an inverted light microscope (EVOS). Five independent fields were assessed for each well, and the average number of tubes per field (magnification, 200x) was determined.

Change in macromolecular permeability of brain ECs was studied by using cell culture transwell insert method [39]. In brief, brain ECs were seeded at a density of 1 × 10^5^ cells/well onto a 24-well transwell insert (pore size, 0.4 μm; polycarbonate membrane, Greiner Bio-One, Germany). The EPCs or NPCs (2 × 10^4^ cells/well) were plated on the lower chamber of the transwell insert system. Then the confluent ECs were subjected to hypoxic culture. During the reoxygenation period, brain ECs were co-cultured with the EPCs and/or NPCs for 24 h. FITC-conjugated dextran (1 mg/ml; 10 k Dalton, Sigma) was added to the upper compartment 90 min before the end of the experiment. The relative fluorescence passed through the polycarbonate membrane into the lower chamber was determined by using a fluorescent plate reader (Synergy, Bio-Tek, Vermont). Relative fluorescent changes to normoxia were presented. ECs in the vehicle group were cultured with EC culture medium only. Likewise, Es was calculated as: Es = (E_EPC + NPC_ − E_EPC_ − E_NPC_) / (E_EPC +_E_NPC_) x 100 %.

### Western blot analysis

H/R-injured ECs were harvested after co-cultured with EPCs and/or NPCs. Proteins were extracted with cell lysis buffer (Thermo scientific) supplemented with complete mini protease inhibitor tablet (Roche). Protein lysates were electrophoresed through SDS-PAGE gels and transferred onto PVDF membranes. The membranes were blocked with 5 % non-fat milk for 1 h and incubated with primary antibodies against Akt (1:1000; Cell Signaling), p-AKt (1:1000; Cell Signaling), VEGFR2 (1:1000; Cell Signaling), p-Flk1 (1:200; Santa Cruz), and $\beta$-actin (1:4000; Sigma) at 4 °C for overnight. After washing, membranes were incubated with horseradish-peroxidase-conjugated IgG (Jackson Immuno Research Lab) for 1 h at RT. Blots were developed with enhanced chemiluminescence developing solutions and quantified under ImageJ software. For detecting the protein expressions of Akt and p-Akt in all groups, two sets of gels were done. One set of gels was used to compare the difference between normoxia and hypoxia groups, and the other set of gels was used to compare the differences among different treatment groups in the Hypoxia groups. All experiments were repeated at least six times. Similarly, Es was calculated as: Es = (E_EPC + NPC_ − E_EPC_ − E_NPC_) / (E_EPC +_E_NPC_) x 100 %.

### ELISA assay of VEGF and BDNF

For determining the baseline level of VEGF and BDNF in the culture medium of EPCs and NPCs, we collected their respective culture medium before the co-culture experiments. After co-culture with EPCs and/or NPCs, the conditional medium of H/R-injured ECs in various groups was also collected. The levels of trophic factors VEGF and BDNF in the culture medium were determined with ELISA kits (R&D systems) by following the manufacturer’s instructions. ECs cultured in normoxia served as a control. ECs in the vehicle group were cultured with EC culture medium only.
